# The GC Skew Index: A Measure of Genomic Compositional Asymmetry and the Degree of Replicational Selection

**Published:** 2007-09-06

**Authors:** Kazuharu Arakawa, Masaru Tomita

**Affiliations:** Institute for Advanced Biosciences, Keio University, Fujisawa, Kanagawa 252-8520, Japan

**Keywords:** GC skew, DNA replication, replicational selection, fast Fourier transforms, bioinformatics, GCSI

## Abstract

Circular bacterial chromosomes have highly polarized nucleotide composition in the two replichores, and this genomic strand asymmetry can be visualized using GC skew graphs. Here we propose and discuss the GC skew index (GCSI) for the quantification of genomic compositional skew, which combines a normalized measure of fast Fourier transform to capture the shape of the skew graph and Euclidean distance between the two vertices in a cumulative skew graph to represent the degree of skew. We calculated GCSI for all available bacterial genomes, and GCSI correlated well with the visibility of GC skew. This novel index is useful for estimating confidence levels for the prediction of replication origin and terminus by methods based on GC skew and for measuring the strength of replicational selection in a genome.

## Introduction

In circular bacterial chromosomes, the replication process starts from a finite replication origin (*ori*) and continues bidirectionally along the two arms (i.e. the replichores) until the replication complex reaches the replication terminus (*ter*), located directly opposite of *ori* (Rocha, 2004a; Rocha, 2004b). Replication is obviously the most fundamental and essential process in the cell cycle of bacteria, and replication also exerts genome-wide mutational and selection pressure, shaping genomic polarity with asymmetrically biased nucleotide composition in leading and lagging strands (Lobry and Louarn, 2003; Lobry and Sueoka, 2002). This compositional skew can be easily observed by plotting the normalized excess of guanine (G) over cytosine (C) content in a subgenomic region with sliding windows along the complete genome sequence (Lobry, 1996). Such a GC skew graph segregates the genome into two regions: one with an excess of G over C corresponding to the leading strand, and the other with an excess of C over G corresponding to the lagging strand. Moreover, the shift points of the GC skew graphs are reportedly correlated with the loci of *ori* and *ter* (Frank and Lobry, 1999). GC skew is observed in many bacterial species with circular chromosomes, although with varying clarity of the shift points, and GC skew is usually not detectable in symbionts and bacteria with linear chromosomes (Worning et al. 2006) or in archaeal genomes, which employ different machinery for the replication process (Grabowski and Kelman, 2003; Lopez et al. 1999; Myllykallio et al. 2000). GC skew is also observed in local genomic regions primarily introduced by RNA synthesis (Fujimori et al. 2005), but the overall genomic polarity due to replication is present regardless of these local effects, and the GC skew is thus observed in intergenic regions as well as in the third nucleotide positions in codons. Although the underlying causes for GC skew is not completely understood, hydrolytic deamination of cytosine in the leading strand in single-stranded state during replication, is suggested as the major contributing factor (Rocha, 2004b).

Because only a few *ori* and *ter* positions had been identified by experimental means, analysis of GC skew was first utilized for the computational prediction of *ori* and *ter* positions by examining available genome sequences (Frank and Lobry, 2000). Similar method using nucleotide gradients of T/C and A/G is utilized for the detection of unidirectional replication in mitochondria (Krishnan et al. 2004; Seligmann et al. 2006). To improve the accuracy of prediction, cumulative diagrams are commonly employed to balance out the noise in sequence composition and to eliminate the requirement for window slides (Grigoriev, 1998), coupled with purine and keto excesses and GC skew (Freeman et al. 1998). However, predictions based on these methods are less accurate in genomes where GC skew cannot be strongly observed (Zawilak et al. 2001). To observe the control of replicational selection on the various genomic properties, genomic compositional skews are also used in conjunction with other genomic features such as the gene orientation (McLean et al. 1998), the distribution of RAG oligomers recognized by the FtsK translocase (Hendrickson and Lawrence, 2006), and the codon bias of genes along the genome (Daubin and Perriere, 2003). To our knowledge, however, no method to quantify the strength of GC skew has been proposed; therefore, it is difficult to compare the effects of replicational selection across bacterial genomes.

In this work, we present the GC skew index (GCSI), which quantifies the strength of GC skew of a given genome by combining Fourier power spectral analysis with the Euclidean distance between the maximum and minimum of the cumulative skew vector. Spectral analysis using fast Fourier transform (FFT) is able to identify the frequency components contributing to a given signal, and it has been applied successfully to the field of bioinformatics (Dodin et al. 2000; Katoh et al. 2002; Yin and Yau, 2005). Because GC skew emerges from the mutational selection in the two replichores, the greatest contributing frequency component of GC skew should be at 1 Hz, with two clear shift points. This observation of a 1-Hz signal combined with the degree of skew calculated by the distance measure between the two vertices of a cumulative skew diagram effectively quantifies the skew of genomic compositional asymmetry.

## Materials and Methods

### Sequences and software

Complete circular chromosomal sequences of 303 bacteria and complete genome sequences of 29 archaeal genomes in GenBank format were selected and obtained from the NCBI RefSeq FTP repository (ftp://ftp.ncbi.nih.gov/genomes/Bacteria/). All analyses were conducted using the G-language Genome Analysis Environment version 1.6.11 (Arakawa et al. 2003; Arakawa and Tomita, 2006). The positional coordinate system for the genomic sequence used in this work was set to originate at 0, unlike that of GenBank, which uses 1 for the position of the first base.

### Calculation of GC skew

GC skew was defined as the normalized excess of C over G in a given sequence, (C − G)/(C + G), which is calculated with sliding windows along the genome. GC skew is defined to be 0 when the amount of C equals that of G. To eliminate the use of window slides, cumulative skew can be calculated as the cumulative sum of the walker graph score at each nucleotide position along the genome, with scores A = 0, T = 0, G = 1, and C = −1. In this work, however, the cumulative GC skew was calculated by taking the cumulative sum of the GC skew in each of the windows, to normalize the cumulative skew strength without it being affected by the length of the genome.

### Fast fourier transform

FFT is the computationally optimized derivation of discrete Fourier transform (DFT) for the number of sampling units in the power of two. FFT transforms a given signal in the time domain to reveal the frequency components comprising the input signal. GC skew can be thought of as a signal along the continuous axis of genomic position, which was used in place of the time domain in this work. DFT *F*(*k*) of a signal of length *N*, *f* (*n*), where *n* = 0, 1, …, *N* − 1, at frequency *k* was calculated as follows:

(1) $$F(k)\;=\;\sum_{n=0}^{N\;-\;1}f(n)e^{-i2\pi kn/N}$$

where *i* = √ 1̅. The power spectrum *PS*(*k*) of *F*(*k*) was further defined as

(2) $$\mathrm{PS}(k)\;=\;|F(k){|}^{2},\;k\;=\;0,\;1,\;2,\;\ldots ,\;N\;-\;1$$

at each frequency *k*. In this power spectrum, GC skew shows the greatest contributing component at 1-Hz frequency, corresponding to the two replichores shifting between two regions of opposite polarity as in a sine curve (Arakawa et al. 2007). The Math:FFT module of Perl (http://search.cpan.org/~rkobes/Math-FFT-1.28/FFT.pm) was used for FFT calculation. To level the effects of genome size when comparing the diverse bacterial species, all genome sequences were divided into 4096 windows, and then the GC skew used as the initial signal, the cumulative GC skew, and the power spectra were calculated in these windows. Number of windows must be the power of two for effective FFT calculation, and here 2^12^ = 4096 windows were used to take account of the effects of gene positioning, since this window size roughly corresponds the size of genes (about 1kbp) in bacterial genomes. This window size also eliminates other local mutational factors including those within genes, generated by functional requirements in RNA synthesis and translation.

### GC skew index

Because cumulative skew should remain around zero under conditions of no strand bias and inversely increase its value in both positive and negative directions where bias is strong, Euclidean distance between the maximal and minimal vertices can be used as a measure of skew. The limitation of this approach and the central challenge for the quantification of genomic compositional skews, however, reside in the mathematical assessment of the skew structure to have exactly two regions physically balanced in length but with opposite polarity of nucleotide content. FFT is a good method for such a purpose, because it is able to reveal the contributing frequency components. Therefore, we used FFT to assess the fitness of the skew to the replicational selection model and combined this with the Euclidean distance between the two vertices of cumulative skew to calculate the GCSI. The GCSI is defined as the normalized average of the Euclidean distance between the two vertices of cumulative skew (*dist*) and the ratio of spectral strength at 1 Hz and the average strength of spectra in frequency regions 2 Hz or above (*SR*). Because the replicational selection is the single most dominant factor for GC strand bias, the ratio of spectral strength at 1-Hz frequency and that of all other spectra or their average must be greater than 1. *SR* was normalized by division with the rounded maximal *SR* of all bacterial genomes, which we defined here as 6000. Likewise, *dist* was normalized by 600.

### Statistical assessment of the significance of GCSI

Significance of the GCSI values is tested using the distribution of GCSI calculated using two sets of randomized data: GCSI calculated using shuffled GC skew, where the window order is randomized using the GC skew values calculated with the original genome sequence, and GCSI calculated using shuffled genome, where the entire nucleotide sequence of the genome is shuffled while conserving the original nucleotide content. Due to calculation costs, statistical test was conducted using 1000 shuffled GC skew and 100 shuffled genome data sets. Distribution of the resulting GCSI values for the randomized data set was firstly tested for its normality using Kolmogorov-Smirnov Lilliefors test, and the significance of the original GCSI value is calculated using the z-score in the distribution of the randomized data set.

## Results

**Figure 1.**
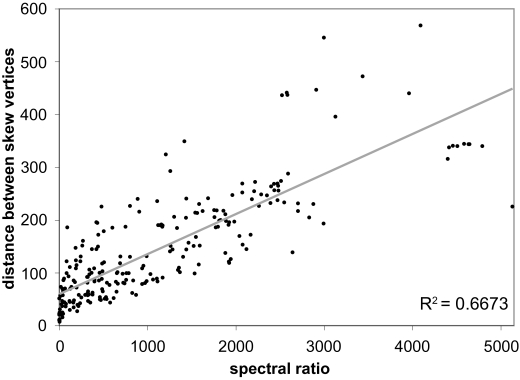
Scatter plot of spectral ratio *RS* against the Euclidean distance between the two vertices in cumulative graph *dist. RS* measures the goodness-of-fit of the “shape” of the overall GC skew to be partitioned into two segments corresponding the two replichores, by calculating the relative predominance of the spectral strength of the 1-Hz frequency component over other frequencies upon applying Fast Fourier Transform. *dist* measures the degree of bias in the leading and lagging strands, by calculating the Euclidean distance between the average GC skew in the two replichores. *RS* is generally correlated with *dist*, therefore combination of these two measures as GCSI should correctly represent both the shape of the graph and the degree of skew.

To test the applicability of GCSI for the quantification of GC skew strength, we first assessed the correlation between the Euclidean distance of the two vertices of cumulative GC skew, *dist*, and the Fourier power spectrum ratio, *SR*, using all genomes (Fig. 1). The two measures correlated with an *R*^2^ value of 0.6673, showing that the predominance of the 1-Hz frequency component leads to a stronger degree of skew.

**Figure 2.**
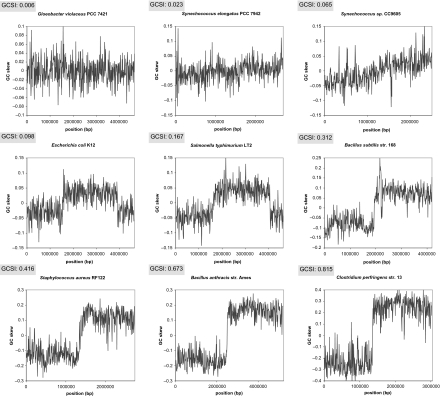
GC skew graphs plotted with 500 windows for nine bacteria at different levels of GCSI. GC skew is not observable for the first two species at GCSI <0.05, and becomes evident at GCSI >0.08. At GCSI >0.1, graphs increase their skewness and the shift points and two replichores can be clearly discerned from the graph. Note that the range of Y-axis extends as GCSI values increase. Overall, GCSI correlates with and correctly captures the degree of skew.

Using the measures *dist* and *SR*, GCSI was calculated for 304 bacterial genomes; 50 selected species are shown in Table 1 (see supplementary information for comprehensive listings). From the comprehensive list, nine genomes were further selected to illustrate the GC skew graphs plotted with 500 windows at various GCSI values (Fig. 2). As a control, GCSI was also calculated for 29 archaeal genomes, most of which showed no GC skew (Table 2). Because GCSI was normalized by the rounded maximum values of *SR* and *dist*, it ranged from 0 to 1. GCSI in bacterial genomes ranged from 0.006 for *Gloeobacter violaceus* to 0.815 for *Clostridium perfringens* (mean, 0.207; median, 0.145; SD, 0.173). The majority of archaeal genomes had GCSI <0.05, and the highest GCSI among archaeal genomes (0.122 of *Halobacterium* sp.) was low compared to those of bacterial genomes. GC skew was not clearly observable in species with GCSI <0.05, but it showed clear shift points when GCSI >0.10. Due to the limited number of iterations, normality test for the statistical assessment using shuffled genome sequence did not score well, but that using shuffled GC skew passed the test in all genome analyzed. The z-score was generally low and therefore not significant when GCSI <0.05 (especially <0.02), where the GCSI values may not be accurate. On the other hand, GCSI >0.05 scored extremely high z-scores, and therefore these values accurately depict the polarity of the genomes.

As can be seen from the GC skew graphs in Figure 2, the degree of skew correlates with GCSI. No skew was observable for *G. violaceus* and *Synechococcus elongatus* PCC 7942, with GCSI values of 0.006 and 0.023, respectively, but a gradual rise from negative values to positive values was observed for *Synechococcus* sp. CC9605, with a GCSI of 0.065, although the skew was not well defined. GC skew became visible at a GCSI of 0.098 in *Escherichia coli* K12, and the clarity was increased in correlation with the GCSI values for scores greater than 1, as represented by the increasing range of the *y*-axis from ±0.15 at GCSI values around 1 to ±0.4 at a GCSI of 0.815.

## Discussion

The nucleotide sequence of a genome is structured and controlled by a myriad of selection pressures, especially in subgenomic regions, as typified by the fact that coding regions are shaped by the essential order and usage of codons. In addition to such requirements in the subgenomic regions, circular bacterial chromosomes experience genome-wide selection through the replication process. The chiral nucleotide composition in the two replication arms is significant; however, with regard to the evolutionary aspects of replicational selection on bacterial chromosomes, no useful method to quantify the degree of genomic compositional asymmetry has been proposed, unlike the wealth of codon bias measures (Suzuki et al. 2005). This lack of indices for genomic compositional skews was likely due to the difficulty of mathematical formulation and detection of the skewing shape of GC skew graphs. To distinguish the degree of skew, we utilized FFT to observe the predominance of the 1-Hz frequency component, which corresponds to the replicational selection on the two replichores, over other frequency components. Combined with the Euclidean distance between the two vertices in cumulative skew graphs, the formulated GCSI captured the strength of GC skew in bacterial chromosomes, as shown by the above results. GCSI scores are diverse even within bacterial genomes with circular chromosomes, ranging from a number of genomes with extremely low values therefore implying the lack of observable GC skew in the genome, to groups of genomes with clear skews as can be seen in Bacilli.

The majority of the archaeal genomes had GCSI <0.05, at which point no noticeable skew is observed even in bacterial genomes. This is also confirmed by the z-score in the statistical test using randomized data, with low z-scores (therefore implying less significance) when GCSI is less than 0.05. Thus, 0.05 can be employed as a threshold value to determine whether GC skew is present in a genome and therefore whether replicational selection is acting on the organism. Because the GCSI values do not show a Gaussian distribution, however, it should be noted that the indices are not necessarily proportionate with each other. Therefore, GCSI values should not be compared in terms of ratios but in terms of their rank orders. For the direct comparison of quantitative degrees of skew calculated as the ratio of two values, the use of Euclidean distance may be more suitable. However, significant Euclidean distance between the two vertices of cumulative skew may not always result from the polarity exhibited by the GC skew graph; it could also result from local regions of highly biased nucleotide content. Therefore, to ascertain that the skews are controlled by replicational selection, genomes used for such analyses should be selected beforehand using GCSI or *SR* at sufficiently high thresholds (e.g. 0.07 for GCSI and 200 for *SR*, also noting the z-scores).

GCSI would be a useful index for the estimation of confidence levels for bioinformatics analyses using genomic compositional skews. Predictions of replication origin and terminus by the observation of shift points (i.e. vertices) of cumulative skew diagrams become erroneous when the GC skew is not well defined. However, the confidence level can be easily estimated by taking into account of the magnitude of the GCSI. In this work we have only described the index for GC skew, although the same method is applicable to purine and keto excesses or any other genomic compositional skews, given that the selection is on the two replichores. Similarly, for comparative studies of genomic features related to evolutionary pressures and replication machinery, GCSI can also be used as a measure of replicational selection.

## Suppliment Material



## Figures and Tables

**Table 1.** GCSI, spectral ratio *RS*, and the Euclidean distance between the two vertices in cumulative graph *dist* for randomly selected 50 bacterial chromosomes. Significance was calculated using 1) 1000 samples by shuffling GC skew windows, and 2) 100 samples by shuffling the entire nucleotide sequence of the genome while conserving the nucleotide composition, and the p-value from the normality test and the significance of the original GCSI value using the distribution of randomized samples was given as the z-score.

							**shuffled GC skew**				**shuffled genome**	

**species**	**accession**	**GCSI**	**SR**	**dist**	**mean**	**SD**	**z-Score**	**p-value (Lillefors)**	**mean**	**SD**	**z-Score**	**p-value (Lillefors)**
*Gloeobacter violaceus PCC 7421*	NC_005125	0.006	2.006	7.103	0.006002	7.88E–05	1	8.08E–62	0.002587	0.000671	5	0.014
*Synechocystis sp. PCC 6803*	NC_000911	0.009	0.296	10.443	0.008784	8.10E–05	0	1.12E–68	0.005882	0.000706	4	0.000
*Mycoplasma hyopneumoniae 232*	NC_006360	0.019	0.149	22.387	0.018741	9.19E–05	0	3.39E–86	0.008964	0.002092	4	0.015
*Synechococcus elongatus PCC 7942*	NC_007604	0.024	35.953	24.632	0.020612	8.28E–05	35	8.91E–63	0.005043	0.000938	19	0.022
*Shigella boydii Sb227*	NC_007613	0.035	120.160	29.756	0.024878	7.99E–05	124	7.41E–67	0.009254	0.000518	49	0.000
*Frankia alni ACN14a*	NC_008278	0.040	125.894	35.514	0.029676	7.46E–05	139	1.95E–54	0.005153	0.000263	132	0.006
*Thiobacillus denitrificans ATCC 25259*	NC_007404	0.042	161.970	34.606	0.028922	8.35E–05	160	2.43E–69	0.003939	0.000703	54	0.923
*Tropheryma whipplei str. Twist*	NC_004572	0.048	9.110	56.698	0.047333	8.84E–05	7	1.78E–79	0.02598	0.000883	24	0.063
*Geobacter sulfurreducens PCA*	NC_002939	0.054	167.963	47.796	0.039911	8.49E–05	163	1.80E–80	0.007165	0.000401	116	0.024
*Rhodopseudomonas palustris BisB5*	NC_007958	0.064	365.258	40.691	0.033991	8.07E–05	376	1.32E–66	0.011625	0.000239	220	0.000
*Polaromonas sp. JS666*	NC_007948	0.071	424.069	43.091	0.035995	8.13E–05	433	7.59E–58	0.00298	0.000621	109	0.003
*Shigella flexneri 2a str. 2457T*	NC_004741	0.077	320.650	60.344	0.050368	8.16E–05	326	4.89E–71	0.008386	0.000448	153	0.000
*Haemophilus influenzae 86–028NP*	NC_007146	0.084	177.860	82.588	0.068907	8.17E–05	180	2.15E–63	0.00523	0.001235	63	0.143
*Mycoplasma genitalium G37*	NC_000908	0.086	103.679	93.248	0.077794	9.01E–05	94	3.10E–78	0.018262	0.002466	27	0.007
*Buchnera aphidicola str. APS* (*Acyrthosiphon pisum*)	NC_002528	0.090	84.815	99.584	0.083067	8.06E–05	86	1.40E–71	0.022281	0.002142	31	0.114
*Helicobacter pylori HPAG1*	NC_008086	0.097	182.961	97.720	0.081517	8.68E–05	174	1.75E–77	0.00802	0.001226	72	0.043
*Escherichia coli K12*	NC_000913	0.098	486.480	69.038	0.057613	8.13E–05	497	1.84E–69	0.004953	0.00069	134	0.000
*Corynebacterium glutamicum ATCC 13032*	NC_006958	0.104	321.871	92.134	0.076862	8.03E–05	333	1.69E–61	0.012113	0.000354	258	0.044
*Helicobacter pylori J99*	NC_000921	0.106	187.288	108.770	0.090726	8.31E–05	186	2.02E–66	0.018694	0.000701	124	0.008
*Rhodospirillum rubrum ATCC 11170*	NC_007643	0.113	763.008	59.247	0.049457	8.74E–05	726	4.72E–77	0.003081	0.000736	149	0.017
*Helicobacter acinonychis str. Sheeba*	NC_008229	0.119	239.480	118.460	0.098801	8.17E–05	243	1.12E–62	0.007669	0.001377	80	0.477
*Escherichia coli O157:H7 str. Sakai*	NC_002695	0.121	662.307	79.123	0.06602	8.36E–05	658	3.89E–68	0.008232	0.000316	356	0.068
*Dehalococcoides sp. CBDB1*	NC_007356	0.127	490.612	102.952	0.085878	8.61E–05	473	1.69E–72	0.010229	0.0009	129	0.144
*Neisseria meningitidis Z2491*	NC_003116	0.138	484.060	117.689	0.098156	8.08E–05	498	7.08E–66	0.004362	0.000929	144	0.129
*Neisseria gonorrhoeae FA 1090*	NC_002946	0.142	508.853	119.737	0.099867	8.29E–05	510	2.72E–60	0.006794	0.001039	130	0.122
*Yersinia pestis KIM*	NC_004088	0.148	785.937	99.533	0.083032	8.47E–05	772	4.61E–63	0.017378	0.00027	485	0.002
*Rickettsia typhi str. Wilmington*	NC_006142	0.161	437.431	149.404	0.124586	8.32E–05	437	9.13E–71	0.048368	0.000704	159	0.533
*Salmonella typhimurium LT2*	NC_003197	0.167	1107.149	89.390	0.074569	7.57E–05	1217	4.49E–65	0.002732	0.000705	232	0.001
*Yersinia pseudotuberculosis IP 32953*	NC_006155	0.174	951.989	113.617	0.094763	8.49E–05	933	8.63E–77	0.017276	0.000232	676	0.054
*Dechloromonas aromatica RCB*	NC_007298	0.199	1366.887	101.487	0.084655	8.43E–05	1350	3.78E–73	0.002758	0.000606	322	0.260
*Chromohalobacter salexigens DSM 3043*	NC_007963	0.220	1568.376	106.845	0.089121	8.36E–05	1561	1.67E–69	0.02062	0.000186	1070	0.000
*Shewanella denitrificans OS217*	NC_007954	0.231	1611.284	116.055	0.096797	8.05E–05	1666	3.01E–60	0.009546	0.000429	515	0.005
*Bacteroides fragilis YCH46*	NC_006347	0.253	1464.908	157.144	0.13104	8.95E–05	1362	3.16E–75	0.003523	0.000726	343	0.046
*Chlamydophila pneumoniae AR39*	NC_002179	0.256	1169.869	189.710	0.158179	8.84E–05	1101	3.49E–72	0.007879	0.00137	180	0.311
*Methylobacillus flagellatus KT*	NC_007947	0.267	1921.680	127.706	0.106502	7.90E–05	2026	5.99E–66	0.005942	0.000789	330	0.057
*Bacillus licheniformis ATCC 14580*	NC_006270	0.271	1879.614	137.460	0.114632	8.87E–05	1764	3.54E–88	0.003241	0.000753	355	0.299
*Desulfotalea psychrophila LSv54*	NC_006138	0.298	2118.652	145.143	0.12104	8.74E–05	2018	1.04E–70	0.034761	0.000188	1393	0.002
*Bacillus subtilis subsp. subtilis str. 168*	NC_000964	0.312	2041.257	169.785	0.141571	8.43E–05	2017	1.59E–71	0.008504	0.000492	615	0.067
*Streptococcus thermophilus LMG 18311*	NC_006448	0.319	1827.666	200.247	0.166958	8.66E–05	1758	6.60E–73	0.015441	0.000843	360	0.245
*Lactococcus lactis subsp*. lactis Il1403	NC_002662	0.329	1753.562	218.954	0.182543	8.30E–05	1759	1.10E–73	0.022	0.000455	673	0.000
*Streptococcus pyogenes SSI-1*	NC_004606	0.332	1881.246	210.648	0.175623	8.53E–05	1836	5.66E–74	0.040869	0.000363	802	0.269
*Streptococcus mutans UA159*	NC_004350	0.378	2260.651	226.932	0.189192	8.33E–05	2259	6.68E–72	0.023889	0.000534	662	0.000
*Bacillus halodurans C-125*	NC_002570	0.406	2702.537	216.866	0.180803	7.92E–05	2841	1.12E–62	0.028966	0.000211	1788	0.643
*Buchnera aphidicola str. Bp (Baizongia pistaciae)*	NC_004545	0.409	1415.720	348.910	0.290845	8.67E–05	1359	3.03E–70	0.011699	0.002443	162	0.005
*Staphylococcus aureus subsp. aureus COL*	NC_002951	0.420	2398.078	263.652	0.219797	8.72E–05	2290	2.05E–70	0.02288	0.000463	857	0.124
*Lactobacillus johnsonii NCC 533*	NC_005362	0.438	2512.013	274.215	0.228599	8.63E–05	2425	8.56E–71	0.038523	0.000349	1142	0.001
*Ehrlichia ruminantium str. Welgevonden*	NC_005295	0.579	2583.741	436.829	0.364108	7.99E–05	2694	6.11E–61	0.047406	0.000605	879	0.837
*Lactobacillus plantarum WCFS1*	NC_004567	0.615	5130.119	225.397	0.187914	8.59E–05	4977	4.07E–76	0.008182	0.000715	848	0.001
*Bacillus anthracis str. Sterne*	NC_005945	0.669	4584.878	344.506	0.287173	8.28E–05	4611	1.26E–62	0.020516	0.000228	2840	0.271
*Clostridium perfringens str. 13*	NC_003366	0.815	4092.849	568.720	0.474015	7.93E–05	4301	1.73E–62	0.108625	0.000249	2832	0.153

**Table 2.** GCSI, spectral ratio *RS*, and the Euclidean distance between the two vertices in cumulative graph *dist* for 29 archaeal chromosomes. See Table 1 legend for the details of the test of significance.

							**shuffled GC skew**				**shuffled genome**	

**species**	**accession**	**GCSI**	**SR**	**dist**	**mean**	**SD**	**z-Score**	**p-value (Lillefors)**	**mean**	**SD**	**z-Score**	**p-value (Lillefors)**
*Thermotoga maritima MSB8*	NC_000853	0.079	55.630	89.074	0.07431	8.28E–05	55	7.84E–73	0.053342	0.000293	87	0.6718442
*Aeropyrum pernix K1*	NC_000854	0.040	3.985	47.125	0.039355	8.41E–05	2	9.80E–70	0.024235	0.000362	42	0.0041011
*Pyrococcus abyssi GE5*	NC_000868	0.045	26.443	51.771	0.043226	8.29E–05	25	4.59E–69	0.012958	0.000897	36	0.172579
*Methanocaldococcus jannaschii DSM 2661*	NC_000909	0.087	14.536	102.413	0.085427	8.30E–05	13	4.54E–70	0.039171	0.000559	84	0.5234979
*Methanothermobacter thermautotrophicus str. Delta H*	NC_000916	0.045	67.648	47.539	0.039698	7.85E–05	70	8.45E–61	0.007545	0.001076	35	0.0004862
*Archaeoglobus fulgidus DSM 4304*	NC_000917	0.020	3.695	23.137	0.019366	8.57E–05	2	1.63E–72	0.012813	0.000474	14	0.0639122
*Pyrococcus horikoshii OT3*	NC_000961	0.105	75.244	117.845	0.098287	8.08E–05	76	1.23E–64	0.043455	0.000316	193	0.5840531
*Thermoplasma acidophilum DSM 1728*	NC_002578	0.046	43.474	50.894	0.042499	8.71E–05	40	2.08E–69	0.014096	0.000693	46	0.0003436
*Halobacterium sp. NRC-1*	NC_002607	0.122	617.687	84.760	0.070717	8.43E–05	609	1.26E–70	0.006544	0.000784	147	0.3059486
*Thermoplasma volcanium GSS1*	NC_002689	0.044	39.413	49.277	0.041153	9.40E–05	33	2.58E–83	0.010706	0.001065	31	0.0006178
*Sulfolobus solfataricus P2*	NC_002754	0.042	17.321	48.204	0.040254	8.11E–05	16	5.41E–62	0.009386	0.000742	43	0.0113624
*Sulfolobus tokodaii str. 7*	NC_003106	0.033	1.862	39.909	0.033345	8.61E–05	0	2.92E–68	0.023109	0.000452	22	0.0383977
*Pyrobaculum aerophilum str. IM2*	NC_003364	0.038	6.535	44.690	0.037329	8.94E–05	5	3.60E–75	0.035224	0.000278	9	0.1593346
*Pyrococcus furiosus DSM 3638*	NC_003413	0.025	0.167	29.587	0.024742	8.62E–05	0	1.43E–69	0.005934	0.00129	14	0.3798953
*Methanopyrus kandleri AV19*	NC_003551	0.023	9.398	26.361	0.022049	8.20E–05	8	1.48E–70	0.01687	0.000478	12	0.0002658
*Methanosarcina acetivorans C2A*	NC_003552	0.012	2.813	13.871	0.011641	7.88E–05	1	7.19E–61	0.003945	0.000539	14	0.1568932
*Methanosarcina mazei Go1*	NC_003901	0.015	2.817	17.149	0.014374	8.09E–05	1	4.87E–64	0.004366	0.000714	14	0.0536714
*Nanoarchaeum equitans Kin4-M*	NC_005213	0.034	3.562	40.488	0.033818	7.74E–05	2	1.39E–66	0.012129	0.002861	7	0.0388642
*Methanococcus maripaludis S2*	NC_005791	0.041	21.214	46.718	0.039014	7.67E–05	21	4.53E–55	0.01073	0.001159	25	0.0074146
*Picrophilus torridus DSM 9790*	NC_005877	0.032	2.862	37.785	0.031568	7.96E–05	1	6.30E–68	0.027789	0.000466	8	0.1271154
*Haloarcula marismortui ATCC 43049*	NC_006396	0.007	5.473	8.170	0.006896	8.40E–05	4	4.38E–62	0.003083	0.000636	6	0.0262528
*Haloarcula marismortui ATCC 43049*	NC_006397	0.027	8.093	31.154	0.026042	7.79E–05	7	7.89E–64	0.017283	0.002185	4	5.38E–05
*Thermococcus kodakarensis KOD1*	NC_006624	0.023	12.060	26.681	0.022315	7.73E–05	11	1.29E–58	0.007225	0.000882	18	0.0146136
*Sulfolobus acidocaldarius DSM 639*	NC_007181	0.036	11.514	41.648	0.034791	8.63E–05	10	9.33E–75	0.006642	0.001216	23	0.2043022
*Methanosarcina barkeri str. fusaro*	NC_007355	0.014	11.210	15.497	0.012995	7.86E–05	10	6.92E–63	0.013765	0.000319	0	8.15E–05
*Natronomonas pharaonis DSM 2160*	NC_007426	0.027	96.855	22.716	0.019012	8.05E–05	99	3.44E–67	0.004615	0.000762	29	1.68E–05
*Methanosphaera stadtmanae DSM 3091*	NC_007681	0.087	85.030	96.236	0.08028	8.38E–05	83	6.57E–71	0.023236	0.000748	85	0.2877452
*Methanospirillum hungatei JF-1*	NC_007796	0.027	17.105	30.715	0.025674	7.94E–05	16	3.41E–72	0.014578	0.000342	36	0.0096521
*Methanococcoides burtonii DSM 6242*	NC_007955	0.044	44.608	48.355	0.040381	8.44E–05	43	1.67E–66	0.029311	0.000319	46	0.0895566
